# The hot spots and trends of Fc gamma receptor: A bibliometric analysis from 2004 to 2024

**DOI:** 10.1097/MD.0000000000042695

**Published:** 2025-06-06

**Authors:** Hui Zhang, Yupeng Wen, Ngit Shin Lai

**Affiliations:** a Institute for Research in Molecular Medicine (INFORMM), Universiti Sains Malaysia, George Town, Pulau Pinang, Malaysia; b College of Medicine, Huanghuai University, Zhumadian, Henan, China.

**Keywords:** bibliometric, CiteSpace, Fc gamma receptor, VOSviewer, web of science core collection

## Abstract

**Introduction:**

FcγR (Fc gamma receptor) is a glycoprotein involved in various biological activities, such as inflammation and tumor immunity, and new ideas about the role of FcγR have also been published recently. Our study utilized journals derived from those published from 2004 to 2024 to analyze the research hotspots and cutting-edge ideas in this field.

**Method:**

All publications were searched using the web of science core collection database. VOSviewer, the R package Biblioshiny in R-studio and CiteSpace (version 6.1.R6) were utilized to perform bibliometric analysis which focused on authors, countries, organizations, keywords, etc.

**Result:**

The analysis of this article is based on 6849 articles related to FcγR from 107 countries and 37,487. The most cited reference in FcγR field is the article “Fcgamma Receptors as Regulators of Immune Responses,” authored by Nimmerjahn. Country/region analysis shows that the United States of America (USA) has far more citation frequency and publications than other countries. The most recent hotspots and keywords are “COVID-19” and “SARS-CoV-2.”

**Discussion:**

Through bibliometric analysis, we can clearly recognize the evolution of the field of FcγR research, from the original cellular immunity to tumor immunity to the occurrence of the latest viral immunity, which may guide the direction of research in the field and allow researchers to be more aware of the current status and frontiers of the field.

## 1. Introduction

FcγR (Fc gamma receptor) is a class of transmembrane glycoprotein that is widely expressed on the surface of immune cells and is a receptor for the IgG constant (Fc) region. It can comprise several subtypes by binding IgG.^[1,2]^ Based on its ability to interact with IgG, FcγRs can be further subdivided into high and low-affinity receptors. Low-affinity FcγRs play an important role in meditating antibody functions in vivo, showing affinities ranging from 30 to 1000 nM, and are widely distributed in monocytes, dendritic cells and macrophages.^[3]^ For example, low-affinity FcγRs could trigger with the Fc, then promoting FcγRs cluster within the cell membrane and cross-link through IgG ICs binding simultaneously to multiple FcγRs, and multiple pathways associating with key cellular functions could be active, like the antibody-dependent cellular cytotoxicity (ADCC), antibody-dependent cellular phagocytosis, and induction of cytokines and chemokines.^[4]^ Immune cells innating is influenced by the ration of activating FcγRs (FcγRIIa [CD32A], FcγRIIIa [CD16A]) and inhibitory FcγRs (FcγRIIb [CD32B]).^[2,5]^ High-affinity FcγR, FcγRI (CD64), occur in monocytes, dendritic cells and macrophages, it could bind monomeric uncomplexed IgG molecules.^[3,6]^ Besides, natural killer cells mainly express FcγRIIIa (CD16).^[7]^ The functions of FcγRs are complex, as the balance between activating FcγRs and inhibitory FcγRs shifts, connecting different signaling pathways and triggering different cellular functions. Previous studies have revealed that Fc-FcγR functional networks are associated with increased susceptibility of cancer patients to bacterial infections, chronic inflammation, and autoimmune diseases such as HIV, as well as with antibody therapy. FcγRs are popular therapeutic research targets, and there is still much potential for their application in various fields.^[8–12]^

Bibliometric analysis is used for qualitative review and analysis with the help of mathematical and statistical techniques in a specific research field over a specific period of time.^[13]^ This method enables the identification and analysis of countries, institutions, keywords, journals, and authors associated with specific research fields, which objectively demonstrate the development trends and the latest progress in the research field.^[14,15]^ Although research on FcγRs has developed rapidly in the past, there is no adequate literature on bibliometric analysis of the progress of FcγRs. Therefore, this study aims to analyze the overall situation of FcγR research through 2 bibliometric softwares, VOSviewer and CiteSpace, and explore the development trends and the latest progress of FcγR research to guide the development direction and showcase the prospects of this field.

## 2. Materials and methods

### 2.1. Bibliometric analysis

Bibliometric analysis is a quantitative methodology employed to assess the academic literature and publications. It applies statistical and mathematical techniques to evaluate the impact, productivity, and trends within a specific field of study or across multiple disciplines.^[14]^ The bibliometric analysis of FcγR was conducted to understand academic literature and research trends, helping researchers, institutions, and policymakers make informed decisions.

### 2.2. Data acquisition

This study did not involve any animals or humans; therefore, ethical approval was not required. The web of science core collection (WOS) is a comprehensive research database that provides access to a wide range of scholarly literature across various disciplines. It is part of the larger WOS platform, that is widely used by researchers, institutions, and libraries for academic research and citation analysis. All literatures were retrieved from “Core Collection databases online” on the WOS and “SCI Expanded (2004-present)” up to November 24, 2024. The search formula was as followed: TS= ([Fcγ receptor*] OR [FcγR*] OR [Fc gamma receptor*] OR [Fcγreceptor*] OR [Fcgamma receptor*] OR [Fcγ-receptor*] OR [FcgammaR*]). Symbol “*” is the retrieval symbol in WOS and represent any 1 character.

### 2.3. Statistical analysis and research methods

In this study, online analysis tools named “Citation Report” and “Results Analysis” in the WOS, VOSviewer (version 1.6.19; Centre for Science and Technology Studies, Leiden University, Leiden, The Netherlands ), R package Biblioshiny in R-studio (version 4.4.0), and CiteSpace (version 6.1.R6; Chaomei Chen, Drexel University, Philadelphia) were utilized to perform bibliometric analysis.

Online analysis tools “citation report” and “results analysis” were used to conduct an initial analysis of the literature retrieved from the WOS database, including the type of literatures, citation times and research categories.

VOSviewer (version 1.6.20) is a software tool used for creating, visualizing, and exploring bibliometric networks such as co-authorship, co-citation, and co-occurrence analysis. This study was supported by the Centre for Science and Technology Studies of Leiden University.^[16]^ In addition to constructing the network, VOSviewer can facilitate the calculation of relevance metrics between items, such as Total Link Strength (TLS).

Biblioshiny is a web-based graphical user interface designed for the R package “bibliometrix” in R-studio (version 4.4.2), facilitating bibliometric analysis and research evaluation. Bibliometrics is an academic discipline that employs quantitative methods to analyze scholarly literature, thereby enabling researchers to evaluate the impact of publications, identify emerging trends in research, and visualize bibliographic data.^[17]^ For example, an overview of the retrieved publications and the impact factors (IFs, h-index, g-index, and m-index) of both the author and journal were collected. Furthermore, the authors’ productivity over time, topic trends and countries production map were visualized.

CiteSpace software (version 6.1.R6) developed by Chen, is a comprehensive software designed for visualizing and analyzing trends and patterns in the scientific literature.^[18]^ Cluster analysis, dual-map overlap analysis, timeline or time zone map and burst map were performed in the CiteSpace tool. Modularity *Q* and the Mean Silhouette value are 2 crucial evaluation metrics in cluster analysis. According to cluster analysis, the results were both significant and reasonable (modularity *Q* > 0.3, mean Silhouette value > 0.7).

## 3. Results

### 3.1. The overview of publication

**Figure 1.**
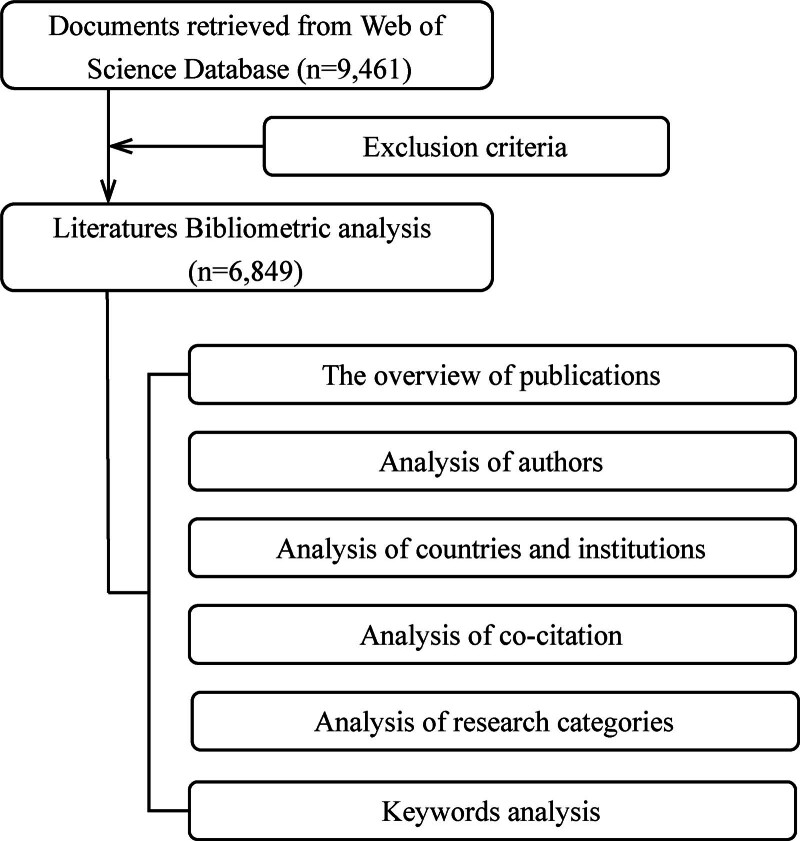
The flowchart of bibliometric analysis.

The process of bibliometric analysis in the field of FcγR is summarized in Figure 1, presented as a flowchart. The retrieval strategy in the WOS database yielded a total of 9471 publications related to FcγR spanning from January 1, 2004, to November 24, 2024. After executing the exclusion criteria, 6849 publications were finally recruited for bibliometric analysis (1425 reviews, 794 meeting abstracts, 106 editorial materials, 106 proceeding papers, 75 letters, 72 book chapters, 40 correction, 12 retracted publications, 3 news items, 1 meeting, 2 prepublications and 28 non-English languages were excluded). The individual authorship of 67 documents were observed among them.

**Figure 2.**
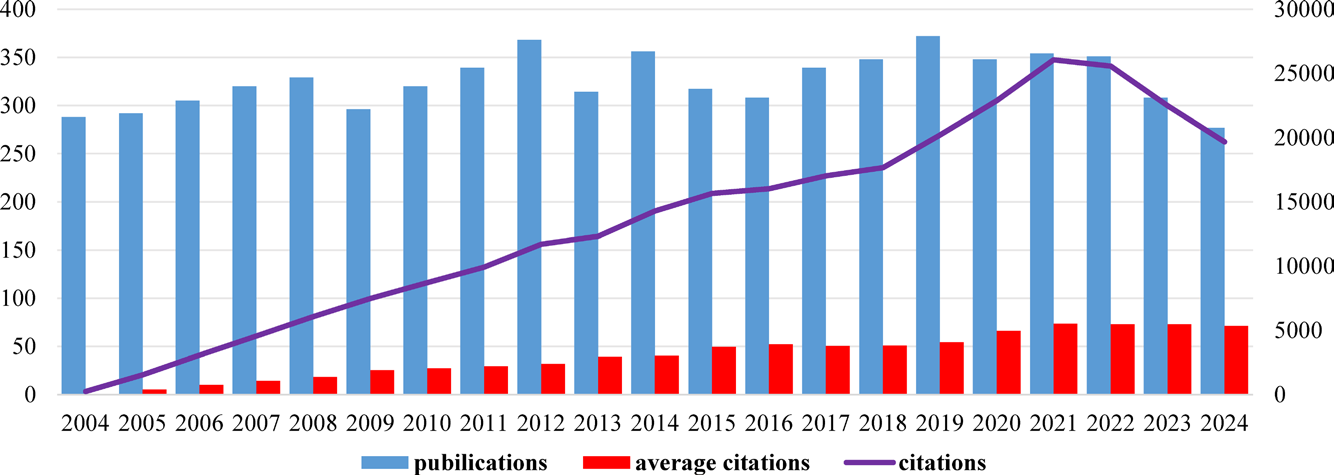
The trends of publications and citations. The x-axis denotes the years. The y-axis denotes the number of publications and secondary y-axis denotes the number of citations. Blue bar represents the publications; red bar represents the average citations; purple line represents the citations.

Overall, as shown in Figure 2, the annual average output of articles pertaining to FcγR surpassed 270 publications. Consequently, the annual growth rate of publications was only 0.05%. Remarkably, the period from 2004 to 2021 witnessed significant and rapid growth in citations. However, in the last 3 years, the citations had exhibited a declining trend over the past 3 years. The average citations per document was 41.39. The average citation showed a consistent annual increase, reaching its peak in 2021.

Table S1, Supplemental Digital Content (https://links.lww.com/MD/P109), illustrates the 10 most cited articles in the field of FcγR. The most frequently cited article, titled “Anti-inflammatory Activity of Immunoglobulin G Resulting from Fc Sialylation,” was authored by Kaneko et al and published in the journal Science in 2006, garnering 1404 citations.^[19]^ Next, literature named “Human lymphoid and myeloid cell development in NOD/LtSz-scid IL2Rγnull mice engrafted with mobilized human hemopoietic stem cells” was cited 1317 times.^[20]^ It was written by Shultz LD et al and published in the Journal of Immunology in 2005. Furthermore, articles with more than 1000 citations also included “Specificity and affinity of human Fcγ receptors and their polymorphic variants for human IgG subclasses” (citations = 1105),^[21]^ “Fc-dependent depletion of tumor-infiltrating regulatory T cells co-defines the efficacy of anti-CTLA-4 therapy against melanoma” (citations = 1094)^[22]^ and “Signaling in Innate Immunity and Inflammation” (citations = 1067).^[23]^

### 3.2. Analysis of author

The total number of authors involved in all publications amounted to 37,487, yielding an average of 5.47 authors per paper. Among these, single-author accounted for 67 documents. The top 10 most productive authors in the field of FcγR are exhibited in Table 1. Nimmerjahn, Ravetch, and Verbeek were among the most representative experts in this field with 90, 74, and 68 publications and 8670, 11,647, and 3326 citations respectively. Relevantly, the significant contribution of Wuhrer should not be disregarded, as evidenced by his 47 treatises that have garnered a total of over 3000 citations (3028). The h-index, g-index, and m-index are quantitative measures employed to assess the scholarly output and citation impact of a researcher’s publications.^[24]^ The researcher Ravetch achieved the highest h-index and m-index values, with scores of 52 and 2.476 respectively. Nimmerjahn gained the highest g-index and with a score of 90. The authors’ production over time was showcased in Figure S1, Supplemental Digital Content, https://links.lww.com/MD/P110. The graph illustrated the publication years and citation counts of the top 10 authors’ works spanning from 2004 to 2024. Dot size is indicative of the number of publications, where color denotes citation counts. The authors, including Nimmerjahn, Ravetch, Leusen and Hogarth have never lost their appeal in the field of FcγR. They had consistently contributed to the study of FcγR development. Nimmerjahn published the most articles (n = 5) and had the highest number of citations in 2012. Additionally, in recent years, there had been a surge of emerging authors such as Alter and Cragg.

**Table 1:** The most productive authors in the field of FcγR, ranked within the top 10.

Authors	Publications	h-index	g-index	m-index	TC	PY-start
Nimmerjahn	90	44	90	2.200	8670	2005
Ravetch	74	52	74	2.476	11,647	2004
Verbeek	68	35	57	1.667	3326	2004
Vidarsson	65	27	53	1.421	2954	2006
Alter	57	27	52	1.688	2741	2009
Cragg	51	26	48	1.733	2401	2010
Leusen	48	23	44	1.095	1949	2004
Wuhrer	47	27	48	1.800	3028	2010
Zhang	45	17	31	1.000	1018	2008
Hogarth	42	21	35	1.000	1261	2004

FcγR = Fc gamma receptor, PY-start = publication year start, TC = total citation.

**Figure 3.**
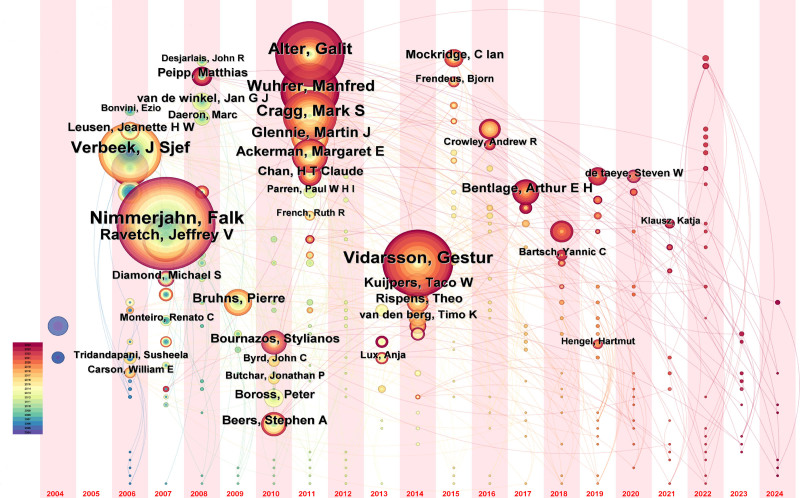
The time zone chart related to the author.

The time zone chart related to the author was generated using the CiteSpace software, as shown in Figure 3. The singular circle symbolizes an individual author, while the line connecting authors represents their correlation. The size of the circle corresponds to the number of citations. The color of the circle indicates the reference year. As depicted in Figure 3, the citation of the author primarily focused on the years 2006, 2007, 2011, and 2014. In 2006, Verbeek citation count experienced a significant surge for the first time. The majority of citations obtained were collected between 2012 and 2014. The citation of an article written by Nimmerjahn and Ravetch firstly boosted in field of FcγR in 2007. The time span during which the articles receive citations was extensive. The authors, including Alter, Wuhrer, Cragg, Glennie, Ackerman et al, achieved a significant increase in citations in 2011. Vidarsson was prominent in 2014 with boosting being cited. His citations were mainly on the period from 2020 to 2024.

### 3.3. Analysis of countries and institutions

There were totally 107 countries making contribution to the study of FcγR. Table 2 elaborates the detail of the top 10 most productive countries. The United States of America (USA) exhibited the highest level of productive, publishing a total of 2855 articles and receiving 157,685 citations. Furthermore, it closely collaborated with other counties (TLS = 1796). Additionally, the TLS of Germany and England were also significantly high, with 836 and 807 respectively. Subsequently, China, ranking as the second most productive country, contributed 849 documents and 17,708 citations. However, with the exception of China, the top 10 most productive countries all had an average of more than 40 citations. A country scientific production map was generated in Figure S2, Supplemental Digital Content, https://links.lww.com/MD/P110. Based on the intensity of the blue color, it is evident that the USA had made the most significant contribution to the field of FcγR research.

**Table 2:** The top 10 countries with the highest productivity in FcγR.

Country	Documents	TC	Average citations	TLS
USA	2855	157,685	55.23	1796
China	849	17,708	20.86	363
Germany	786	34,393	43.76	836
Japan	687	27,871	40.57	412
England	605	27,836	46.01	807
Netherlands	538	24,800	46.10	608
France	397	19,467	49.04	432
Canada	328	14,348	43.74	343
Sweden	257	12,513	48.69	317
Switzerland	251	11,712	46.66	384

FcγR = Fc gamma receptor, TC = total citation, TLS = total link strength.

**Figure 4.**
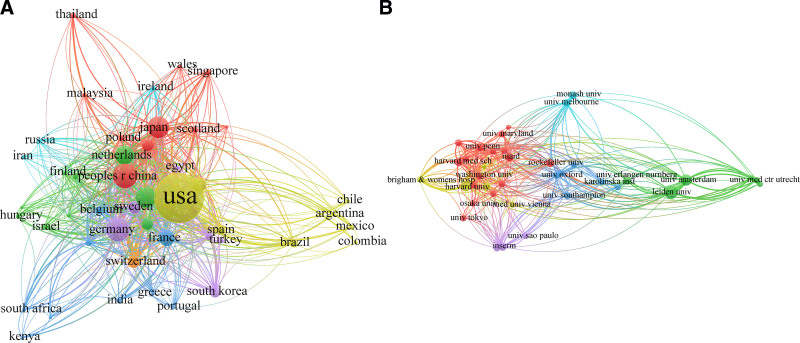
The co-authorship network visualization maps. (A) Co-authorship of countries; (B) co-authorship of institutions.

The co-authorship network visualization map illustrating countries (n = 47) that have published more than 10 documents is presented in Figure 4A. The dot size on this map represents a country’s publication count, while line thickness indicates the level of TLS between countries. The countries were divided into 7 clusters based on K-means algorithm. The countries with the most publications in each cluster were China (cluster 1), England (cluster 2), France (cluster 3), the USA (cluster 4), Germany (cluster 5), Ireland (cluster 6), and Switzerland (cluster 7).

Furthermore, coauthor analysis was employed to investigate institutional collaboration. As shown in Figure 4B, institutions which launched more than 49 documents were brought into coauthor analysis. Ultimately, a total of 38 institutions met the requirements and were categorized into 6 clusters. Leiden University secured the top position in the article ranking with 182 publications in cluster 2 (TLS = 121), followed by University of Amsterdam with 146 publications in cluster 2 (TLS = 98), and Harvard University with 140 publications in cluster 4 (TLS = 110) (Table 3).

**Table 3:** The top 10 coauthor institutions in FcγR.

Institution	Publications	TC	Average citations	TLS
Leiden University	182	9556	52.51	121
University of Amsterdam	146	5981	40.97	98
Harvard university	140	11,385	81.32	110
National Institutes of Allergy and Infectious Diseases	103	7079	68.73	52
Harvard Medical School	99	3370	34.04	84
Institut national de la santé et de la recherche médica	99	7215	72.88	68
University of Pennsylvania	97	5731	59.08	64
University of Toronto	93	4828	51.91	40
Rockefeller University	92	14,012	152.30	53
University of Erlangen–Nuremberg	92	5935	64.51	44

FcγR = Fc gamma receptor, TC = total citation, TLS = total link strength.

**Figure 5.**
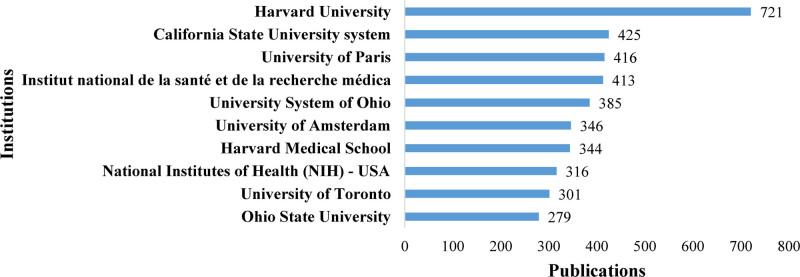
The top 10 most prolific publishing institutions.

Among the institutions with the highest number of publications from 6849 literatures, Harvard University ranked first with 721 publications (Fig. 5). Following this, the California State University system ranked second with 425 publications, while the University of Paris ranked third with 416 publications.

### 3.4. Analysis of co-citation for sources and references

There were totally 1096 sources across 6849 articles, of which the most publication was Journal of Immunology with 466 documents (Table 4). The h-index, g-index, and m-index for this journal are 84, 129, and 4, respectively. Other high productivity sources included Frontiers in Immunology (documents = 282), Blood (documents = 195), PLoS One (documents = 182), and Journal of Biological Chemistry (documents = 140). The IF serves as a metric for assessing the relative significance of a scientific journal within its specific domain. The highest IF among the top 10 most productive journals in the field of FcγR research was Blood (IF = 21). The second-place journal was Proceedings of the National Academy of Sciences of the United States of America (IF = 9.4).

**Table 4:** The top 10 most productive journals in the field of FcγR research.

Journals	Documents	h-index	g-index	m-index	TC	TLS	IF (2023)
Journal of Immunology	466	84	129	4	26,263	953,088	3.6
Frontiers in Immunology	282	37	50	2.643	4719	177,240	5.7
Blood	195	73	120	3.476	16,642	541,203	21
PLoS One	182	39	58	2.167	4961	148,580	2.9
Journal of Biological Chemistry	140	41	66	1.952	5854	415,313	4
Proceedings of the National Academy of Sciences of the United States of America	116	65	112	3.095	12,570	428,676	9.4
mAbs	106	36	60	2.25	3953	73,413	5.6
European Journal of Immunology	103	33	57	1.571	3717	183,599	4.5
Scientific Reports	100	29	43	2.071	2362	57,425	3.8
Journal of Leukocyte Biology	91	36	63	1.714	4309	112,448	3.6

FcγR = Fc gamma receptor, IF = impact factor, TC = total citation, TLS = total link strength.

**Figure 6.**
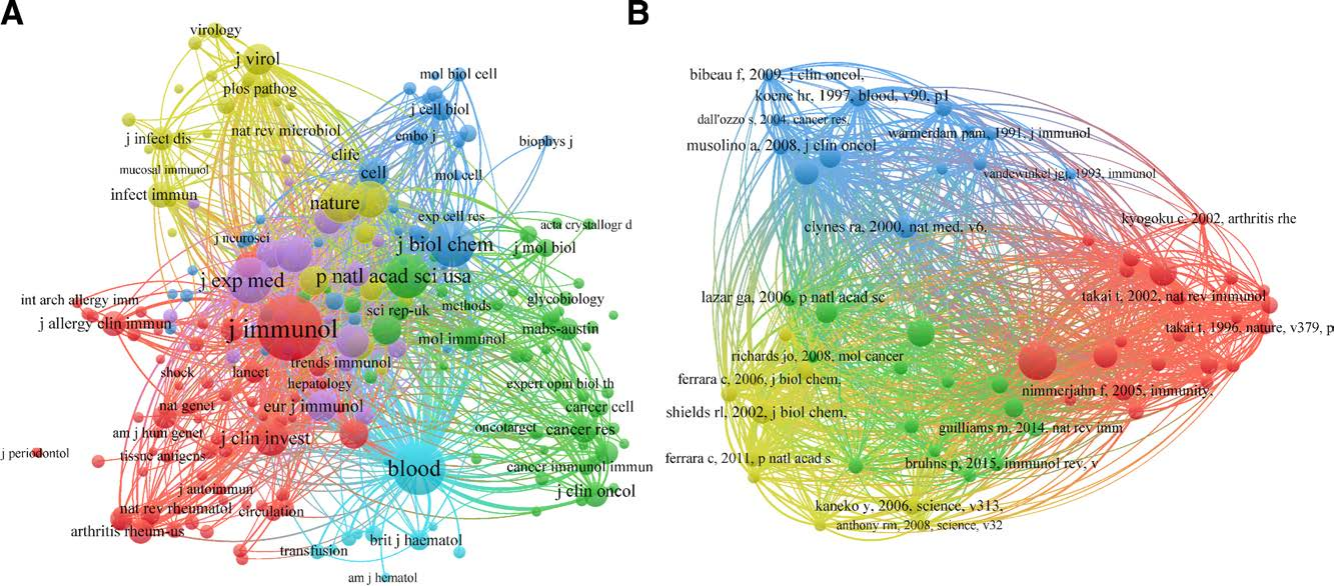
The co-citation network visualization maps. (A) Co-citation of sources; (B) co-citation of references.

Co-citation analysis is a bibliometric method used to evaluate the relationship between academic papers based on their citations, which helps understand the structure of academic literature, identify research trends, and map the intellectual landscape of a specific field. The co-citation analysis of sources and references are visualized in Figure 6. Figure 6A elaborates the co-citation analysis of sources with over 200 citations per journal. Consequently, 220 journals fulfilled the criteria and were classified into 6 clusters. The most frequently co-cited journal in each cluster included Journal of Immunology in cluster 1 (citations = 26,263, TLS = 953,088), Proceedings of the National Academy of Sciences of the United States oF America in cluster 2 (citations = 12,570, TLS = 428,676), Journal of Biological Chemistry in cluster 3 (citations = 5854, TLS = 415,313), Nature in cluster 4 (citations = 6959, TLS = 330,780), Journal of Experimental Medicine in cluster 5 (citations = 10,132, TLS = 456,915) and Blood in cluster 6 (citations = 16,642, TLS = 541,203). The Journal of Immunology was the most extensively cooperative study in the field of FcγR.

Top 10 most frequently cited references in the field of FcγR. As shown in Table S2, Supplemental Digital Content, https://links.lww.com/MD/P109, the most cited reference was the article “Fcgamma Receptors as Regulators of Immune Responses,” authored by Nimmerjahn et al, which was published in Nature Reviews Immunology in 2008.^[2]^ This publication has received 890 citations and a TLS score of 2562. Other frequent references included “IgG Fc receptors”^[25]^ (citation = 489, TLS = 1360) and “Specificity and affinity of human Fcgamma receptors and their polymorphic variants for human IgG subclasses”^[21]^ (citation = 469, TLS = 1750). The top 25 references with the strongest citation bursts were generated with the help of CiteSpace software and were illustrated in Figure S3, Supplemental Digital Content, https://links.lww.com/MD/P110. The article “Fcgamma Receptors as Regulators of Immune Responses” as the most popular reference, experienced a surge in popularity from the beginning of 2009 to the end of 2013 with a strength of 108.32. However, none of the reference burst lasted longer than 5 years.

A co-citation network visualization map of the references is presented in Figure 6B, with a minimum citation threshold established at 100. The co-citation of reference map was categorized into 4 clusters. Cluster 1 comprised 59 items labeled in red, with the most frequently cited article being “Fcgamma Receptors as Regulators of Immune Responses.”^[2]^ The highest citations of reference in cluster 2 (tagged with green) was authored by Bruhns et al and was published in Blood.^[21]^ Cluster 3 was designated with the color blue and comprised 10 items, with the most significant contribution originating from the Blood and authored by Guillaume et al.^[26]^ The reference titled “Lack of fucose on human IgG1 N-linked oligosaccharide improves binding to human Fcgamma RIII and antibody-dependent cellular toxicity” was the most cited in cluster 4 (indicated by year color), with a citation count of 329 and a TLS score of 1793.^[27]^

### 3.5. Analysis of research categories

**Figure 7.**
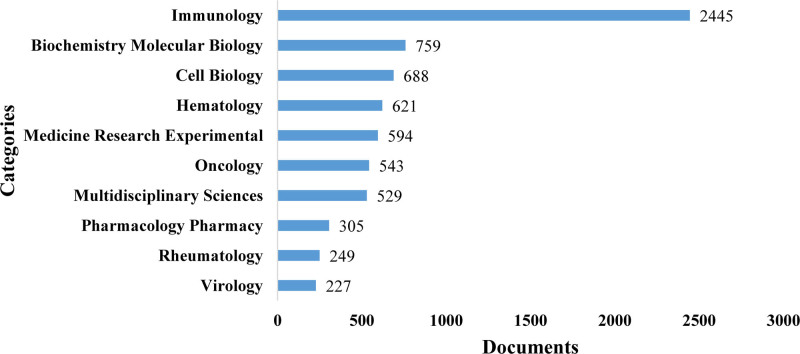
The top 10 research categories with the most articles.

This study encompassed a total of 101 research categories, covering a wide range of research areas. As shown in Figure 7, the top 10 research categories with the most articles contained “Immunology” (2445 articles), “Biochemistry Molecular Biology” (759 articles), “Cell Biology” (688 articles), “Hematology” (621 articles), “Medicine Research Experimental” (594 articles), “Oncology” (543 articles), “Multidisciplinary Sciences” (529 articles), “Pharmacology Pharmacy” (305 articles), “Rheumatology” (249 articles) and “Virology” (227 articles).

**Figure 8.**
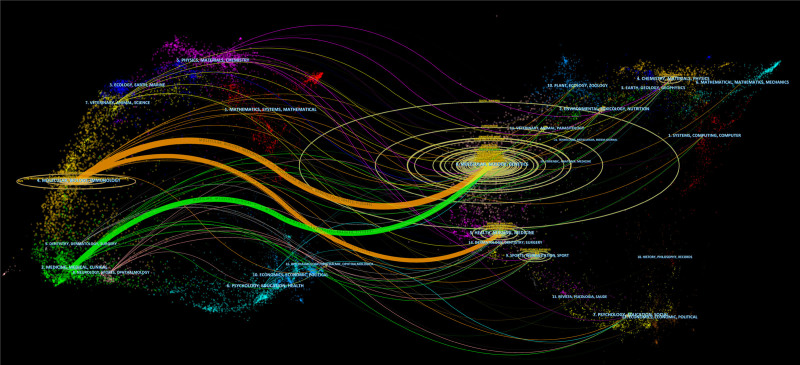
The dual-map overlap map.

Dual-map overlay analysis is a bibliometric methodology employed to illustrate the interrelationships among various research domains and discern trends, linkages, and seminal contributions within these areas.^[28]^ As shown in Figure 8, the left side illustrates the categories of citing journals, while the right side illustrates the categories of cited journals. They are connected to each other by a colorful curve that marks the reference trajectory. Figure 8 displays 3 distinct curves, 2 of which are orange and 1 is green. The category of citing journal primarily including “molecular, biology, immunology” and “medicine, medical, clinical.” The category of citied journal was mainly focus on “molecular, biology, genetics” and “health, nursing, medicine.” Among these, cited journals originating from the fields of “molecular, biology, genetics” constituted the majority.

### 3.6. Keywords analysis

**Figure 9.**
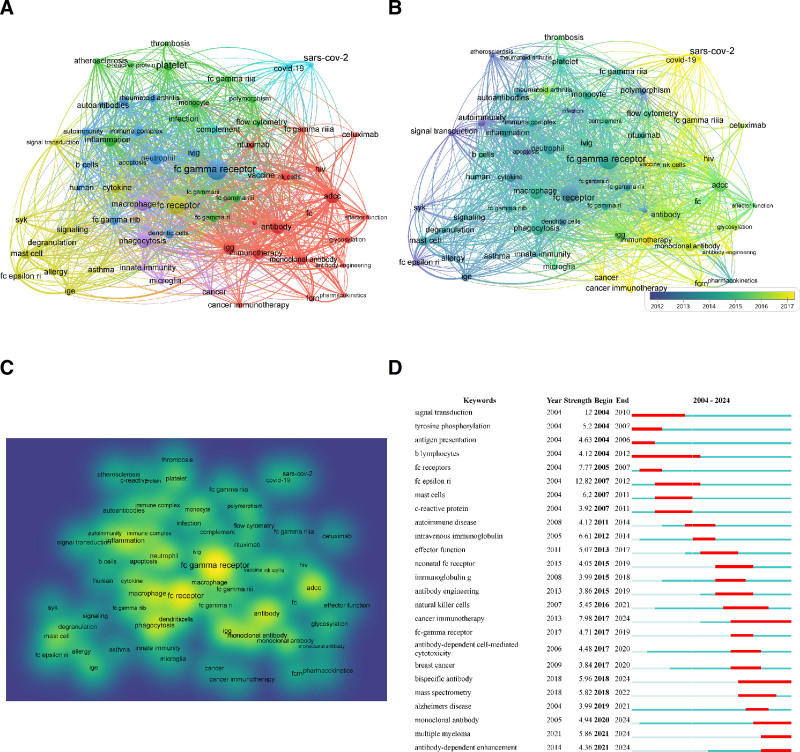
Keyword analysis. (A) Network visualization map; (B) overlay visualization map; (C) density visualization map; (D) top 25 keywords with the strongest citation bursts.

Keywords analysis involves the systematic identification and assessment of keywords to enhance content optimization for search engines, thereby improving visibility and relevance in search results. According to the results of keywords analysis of 6849 publications, 64 keywords exceeding 30 times were included in the analysis. With the assistance of VOSviewer software, network visualization map, overlay visualization map and density visualization map for keywords co-occurrence analysis are generated in Figure 9. Based on the results of network visualization map (Fig. 9A), the 64 keywords were categorized in to 6 clusters. The diameter of each circle is proportional to the frequency of the corresponding keyword, whereas the thickness of each line reflects the degree of correlation between keywords. The most frequent keyword in each cluster was “ADCC (occurrence = 215, TLS = 323)” in cluster 1, “inflammation (occurrence = 189, TLS = 246)” in cluster 2, “fc gamma receptor (occurrence = 426, TLS = 542)” in cluster 3, “fc receptor (occurrence = 325, TLS = 472)” in cluster 4, “macrophage (occurrence = 325, TLS = 472)” in cluster 5, “complement (occurrence = 64, TLS = 131)” in cluster 6. Additionally, other high occurrence keywords contained “phagocytosis (occurrence = 208, TLS = 325, cluster 5),” and “Igg (occurrence = 177, TLS = 296, cluster 1).” Figure 9B indicates the occurrence time of keywords spanning from 2004 to 2024. The majority of the keywords primarily emerged in 2012 to 2015, like “fc gamma receptor,” “fc receptor” and “inflammation.” Other late-emerging keywords included “ADCC,” “antibody” and “immunotherapy.” A density visualization map was designed to illustrate the volume of literature associated with specific keywords. As shown in Figure 9C, the closer the color is near to yellow, the higher the weight of the keyword. The top 25 keywords exhibiting the most significant citation bursts were visualized using the CiteSpace software (Fig. 9D). Most of keywords burst began in 2004 (4/25) and 2017 (4/25), such as “fc receptors (strength = 7.77)” and “antibody-dependent cell-mediated cytotoxicity (strength = 4.48).” The strongest keyword burst was “fc epsilon ri” with a strength of 12.82. The keyword exhibiting the longest burst time span was “B lymphocytes,” which lasted for a period of 8 years. Notably, it was anticipated that the keyword trends concerning “cancer immunotherapy,” “bispecific antibody,” “monoclonal antibody,” “multiple myeloma” and “antibody-dependent enhancement” would persist into 2025, underscoring their enduring relevance as key subjects within the field of FcγR. Moreover, Figure S4, Supplemental Digital Content, https://links.lww.com/MD/P110 further illustrated the evolving trend of keywords, with recent focal points being “COVID-19” and “SARS-CoV-2.”

**Figure 10.**
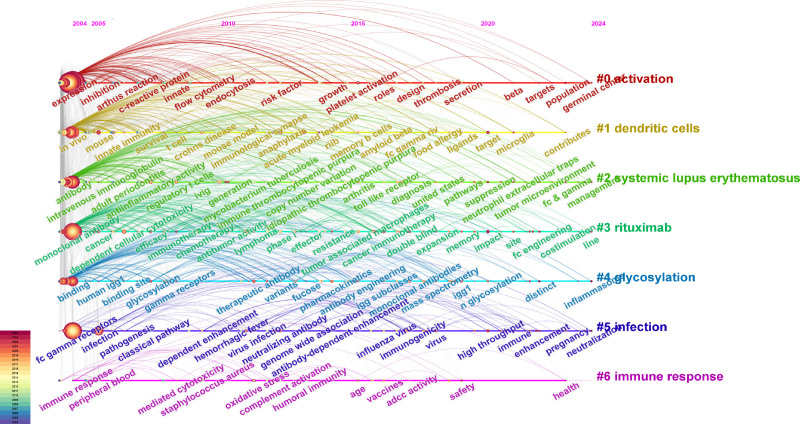
Timeline map of keyword.

The timeline of keyword was plotted with the help of CiteSpace software (Fig. 10) and divided into 7 clusters. The cluster of timelines included “activation,” “dendritic cells,” “systemic lupus erythematosus,” “rituximab,” “glycosylation,” “infection” and “immune response,” in which the most important areas were “activation” and “infection.”

## 4. Discussion

The analysis of this article is based on 6849 articles related to FcγR from 107 countries and 37,487 authors in the WOS database spanning from January 1, 2004 to November 24, 2024. Overall, the number of articles and citations increased from 2004 to 2024, however, in the past 3 years, the number of citations had exhibited a declining trend.

The country/region analysis shows that the USA has far more citation frequency and publications than other countries, and the USA was also the center in FcγR field based on its strongest cooperation with other countries. It was proven that the USA became the most influential country in the field of FcγR. Moreover, most of the top institutions in this field are from the USA, and of the top 3 institutions with the largest amounts of publications, the first and second are both USA-origin institutions, such as Harvard University and California State University. In addition to the States of America, China, England, France, Germany, Ireland and Switzerland contributed significantly to the improvement of FcγR field with a high number of publications and citation frequency.

The author analysis shows that Ravetch and Nimmerjahn may be the most Influential authors based on the h-index, g-index, and m-index. The researcher Ravetch JV achieved the highest h-index and m-index values, with scores of 52 and 2.476 respectively. Nimmerjahn gained the highest g-index with a score of 90. Besides, the most cited reference in FcγR field is the article “Fcgamma Receptors as Regulators of Immune Responses,” authored by Nimmerjahn, Verbeek and Wuhrer had significant contribution to FcγR field, Verbeek has published a total of 68 articles, which have been cited a total of 3326 times, and Wuhrer owns 47 treatises which have garnered a total of over 3000 citations (3028).

Analysis of journals shows that Journal of Immunology, which ranks the highest in the number of publications, and The New England Journal of Medicine, Journal of Clinical Oncology, and accounts for IF rankings in the FcγR field, the 2 top Journals are Blood and Proceedings of the National Academy of Sciences of the United States of America. The most frequently co-cited journals included Journal of Immunology, Proceedings of the National Academy of Sciences of the United States of America, Journal of Biological Chemistry, Nature, Journal of Experimental Medicine and Blood. It could be seen Journal of Immunology was the most extensively cooperative in the field of FcγR.

Keywords analysis is helpful in understanding the frontiers and hotspots of FcγR. In existing studies, the most frequent keyword includes “ADCC,” “inflammation,” “fc gamma receptor,” “fc receptor,” “macrophage,” and “complement.” These are related to the mechanism of FcγR activation and the cells associated with its activation. The majority of the keywords primarily emerged in 2012 to 2015, like “fc gamma receptor,” “fc receptor” and “inflammation.” Most keywords bursts began in 2004 and 2017, and the keyword exhibiting the longest burst time span was “B lymphocytes,” which are one of the most important key cells of FcγR, with lasted for a period of 8 years. Notably, it was anticipated that the keyword trends concerning “cancer immunotherapy,” “bispecific antibody,” “monoclonal antibody,” “multiple myeloma,” and “antibody-dependent enhancement” would persist into 2025, underscoring their enduring relevance as key subjects within the field of FcγR and indicating FcγR is beginning to be gradually applied to tumor immunotherapy, and its potential in this field is gradually being explored. Based on the trend of keywords evolution, the most recent hotspot is “COVID-19” and “SARS-CoV-2,” by article review, it suggested that FcγR involved in the development and treatment of a wide range of infections, and although activation of Fc-FcγR has been shown to inhibit the development of infections, if inappropriately over-activated, it will promote the development of viral infections.^[29]^

The family of FcγRs, given their pivotal role in regulating humoral tolerance, antibody-mediated effector functions, and cellular immune responses, have been recognized as key regulators in modulating both pro-inflammatory and anti-inflammatory responses.^[2]^ However, its function is not limited to the immune system, but is also involved in regulating the physiological and pathological processes of other systems. For instance, in the hematopoietic system, FcγRIIA, serving as the second receptor on human platelets, targets platelet surface glycoproteins, primarily glycoprotein IIb-IIIa (GP IIb-IIIa) and GPIb-IX. This interaction leads to Fc-dependent clearance mediated by macrophages, ultimately resulting in immune thrombocytopenia (ITP).^[30]^ Moreover, recent studies have demonstrated the existence of FcγRs in the central nervous system, indicating their potential involvement in neuroinflammation and neurodegenerative diseases. Normally, IgG antibodies can potentially influence normal neurodevelopment or function through direct interaction with FcγR on nonimmune cells in the brain.^[31]^ Pathologically, recent research has unveiled the potential involvement of the immune system in the pathophysiology of neuropathic pain, particularly focusing on FcγRs. Liu et al demonstrated that the neuronal C-reactive protein/FcγRI positive feedback pro-inflammatory signaling pathway plays a significant role in nerve injury-induced neuropathic pain.^[32]^ Additionally, Jiang et al and Lacagnina et al further validated the involvement of the B cell–IgG–FcγR axis in the pathogenesis of neuropathic pain.^[33,34]^ In the musculoskeletal system, FcγRs are implicated in autoimmune conditions such as rheumatoid arthritis. FcγR-IgG complexes bound to cartilage, specifically targeting collagen type II (CII) or cartilage oligomeric matrix protein, elicit mechanical hypersensitivity in mice, thereby exacerbating joint inflammation and destruction.^[35]^ Advances in our understanding of FcγR biology have opened new avenues for therapeutic interventions, promising to enhance the efficacy of existing treatments and develop novel strategies for managing infectious diseases and cancers. Recent studies have verified the polymorphism of the FcγR gene affecting the efficiency of antibody-mediated pathogen clearance, like HIV-1.^[36]^ Regarding their application in cancer research, their ability to mediate ADCC, enhance phagocytosis, and modulate immune responses positions them as a critical focus in the field. For instance, in patients with breast cancer who are human epidermal growth factor receptor 2 (HER2) positive, Trastuzumab effectively suppresses tumor growth by binding to FcγRIIIa, thereby activating natural killer cells and macrophages and enhancing the ADCC response.^[37]^ A deeper understanding of FcγR biology may pave the way for innovative strategies to modulate immune responses and enhance therapeutic efficacy in diverse clinical scenarios.

To the best of our knowledge, no bibliometric analysis of FcγR research has been conducted prior to this study. Aiming to analyze the overall situation of FcγR research, we utilized bibliometric software, VOSviewer and CiteSpace to identify hotspots and collaborations between authors, countries, facilities and keywords in this area. However, our study had some limitations and shortcomings. First, the journals that we mainly analyzed originated from the English language, and literature in other languages, such as Japanese and Chinese, which were not involved in the analysis of this study. Therefore, the findings of this article may not be applicable to studies published in other languages.^[38]^ Second, because it is difficult for scientometric software to directly combine results from multiple databases, based on the frequency of use of WOS databases in bibliometric analyses and the useful functions such as “create citation report” and “analyze results,” we only utilized WOS to analyze the results of this article during our search. WOS was used in our search, without combining data from PubMed, Scopus, Embase, and other databases. In addition, only the first author was considered in the co-citation analysis with the help of the VOSviewer software, while none of the other authors were included. Moreover, some of the literature has been published for a long time, and the ideas contained in it will be backward, resulting in some bias in the analysis. Furthermore, the literature analyzed in our article was mainly published from January 1, 2004 to November 24, 2024, and 2024 was not over and there were still some articles published there. Publications and the average number of citations per year in 2024 were not included in the annual scientific reports. Overall, hotspots and trends in the FcγR field can still be optimized.

## 5. Conclusion

This study presents a bibliometric analysis of papers published between 2002 and 2024 on the field of FcγR. The United States plays a leading role in this field and collaborates closely with several countries. The 3 most productive and influential journals are Journal of Immunology, Blood and Proceedings of the National Academy of Sciences of the United States of America. Ravetch and Nimmerjahn may be the most Influential authors based on the h-index, g-index, and m-index. A hot research topic in the field of FcγR current is the multiple effects of the Fc-FcγR cascade reaction in viral immunity. These findings will help researchers to recognize the current status of research and research trends in the field of FcγR.

## Acknowledgments

This study was supported by the Shenzhen Longgang District Science and Technology Innovation Special Fund.

## Author contributions

**Conceptualization:** Ngit Shin Lai.

**Data curation:** Hui Zhang, Yupeng Wen.

**Formal analysis:** Hui Zhang, Yupeng Wen.

**Investigation:** Hui Zhang, Yupeng Wen.

**Methodology:** Hui Zhang.

**Project administration:** Ngit Shin Lai.

**Supervision:** Ngit Shin Lai.

**Validation:** Hui Zhang.

**Visualization:** Hui Zhang.

**Writing – original draft:** Hui Zhang, Yupeng Wen.

**Writing – review & editing:** Hui Zhang, Yupeng Wen, Ngit Shin Lai.

## Supplementary Material


