# Supplementary material for: The hot spots and trends of Fc gamma receptor: A bibliometric analysis from 2004 to 2024
Source: Medicine (Baltimore). 2025 Jun 6;104(23):e42695. doi: 10.1097/MD.0000000000042695 (PMC12150913; doi:10.1097/MD.0000000000042695)
Supplement: Supplementary file 1 [file medi-104-e42695-s001.pdf]

Supplementary Table 1 The 10 most cited articles in the field of FcγR.

Article	Author	Journal	DOI	Citations	Year
Anti-inflammatory activity of immunoglobulin G resulting from Fc sialylation	Kaneko Y et al.	Science	10.1126/science.1129594	1,404	2006
Human lymphoid and myeloid cell development in NOD/LtSz-scid IL2Rγnull mice engrafted with mobilized human hemopoietic stem cells	Shultz LD et al.	JOURNAL OF IMMUNOLOGY	10.4049/jimmunol.174.10.6477	1,317	2005
Specificity and affinity of human Fcγ receptors and their polymorphic variants for human IgG subclasses	Bruhns P et al.	BLOOD	10.1182/blood-2008-09-179754	1,105	2009
Fc-dependent depletion of tumor-infiltrating regulatory T cells co-defines the efficacy of anti-CTLA-4 therapy against melanoma	Simpson Tyler R. et al.	JOURNAL OF EXPERIMENTAL MEDICINE	10.1084/jem.20130579	1,094	2013
Signaling in Innate Immunity and Inflammation	Newton Kim et al.	Cold Spring Harbor Perspectives in Biology	10.1101/cshperspect.a006049	1,067	2012
A phase I study on adoptive immunotherapy using gene-modified T cells for ovarian cancer	Kershaw Michael H. et al.	CLINICAL CANCER RESEARCH	10.1158/1078-0432.CCR-06-1183	970	2006
A single-cell atlas of the peripheral immune response in patients with severe COVID-19	Wilk Aaron J. et al.	NATURE MEDICINE	10.1038/s41591-020-0944-y	931	2020
Immunoglobulin G fragment C receptor polymorphisms and clinical efficacy of trastuzumab-based therapy in patients with	Musolino Antonino et al.	JOURNAL OF CLINICAL ONCOLOGY	10.1200/JCO.2007.14.8957	811	2008

HER-2/neu-positive metastatic breast cancer					
Human intestinal macrophages display profound inflammatory anergy despite avid phagocytic and bacteriocidal activity	Smythies LE et al.	JOURNAL OF CLINICAL INVESTIGATION	10.1172/JCI200519229	710	2005
Human lupus autoantibody-DNA complexes activate DCs through cooperation of CD32 and TLR9	Means TK et al.	JOURNAL OF CLINICAL INVESTIGATION	10.1172/JCI200523025	674	2005

Supplementary Table 2 The top 10 most frequently cited references in the field of FcγR.

Article	Author	Journal	DOI	Type	Citations	Year	TLS
Fcgamma receptors as regulators of immune responses	Falk Nimmerjahn et al.	NATURE REVIEWS IMMUNOLOGY	10.1038/nri2206	Review	890	2008	2,562
IgG Fc receptors	J V Ravetch et al.	Annual Review of Immunology	10.1146/annurev.immunol.19.1.275	Review	489	2001	1,360
Specificity and affinity of human Fcgamma receptors and their polymorphic variants for human IgG subclasses	Pierre Bruhns et al.	BLOOD	10.1182/blood-2008-09-179754	Original research	469	2009	1,750
Therapeutic activity of humanized anti-CD20 monoclonal antibody and polymorphism in IgG Fc receptor FcgammaRIIIa gene	Guillaume Cartron et al.	BLOOD	10.1182/blood.v99.3.754	Clinical Trial	395	2002	1,966
Fcgamma receptors: old friends and new family members	Falk Nimmerjahn et al.	Immunity	10.1016/j.immuni.2005.11.010	Review	372	2006	1,216
Lack of fucose on human IgG1 N-linked oligosaccharide improves binding to human Fcgamma RIII and antibody-dependent cellular toxicity	Robert L Shields et al.	JOURNAL OF BIOLOGICAL CHEMISTRY	10.1074/jbc.m202069200	Original research	329	2002	1,793
Inhibitory Fc receptors modulate in vivo cytotoxicity against tumor targets	R A Clynes et al.	NATURE MEDICINE	10.1038/74704	Original research	321	2000	1,394
Two immunoglobulin G fragment C receptor polymorphisms independently predict response to rituximab in patients with follicular lymphoma	Wen-Kai Weng et al.	JOURNAL OF CLINICAL ONCOLOGY	10.1200/jco.2003.05.013	Original research	316	2003	1,697
High resolution mapping of the binding site on human IgG1 for Fc gamma RI, Fc gamma RII, Fc gamma RIII, and FcRn and	R L Shields et al.	JOURNAL OF BIOLOGICAL CHEMISTRY	10.1074/jbc.m009483200	Original research	306	2001	1,514

design of IgG1 variants with improved binding to the Fc gamma R							
FcγRIIIa-158V/F Polymorphism Influences the Binding of IgG by Natural Killer Cell FcγRIIIa, Independently of the FcγRIIIa-48L/R/H Phenotype	Koene Harry R et al.	BLOOD	10.1182/blood.v90.3.1109.1109_1109_1114	Original research	286	1997	1,166

TLS: total link strength
