# Supplementary material for: The hot spots and trends of Fc gamma receptor: A bibliometric analysis from 2004 to 2024
Source: Medicine (Baltimore). 2025 Jun 6;104(23):e42695. doi: 10.1097/MD.0000000000042695 (PMC12150913; doi:10.1097/MD.0000000000042695)

**Supplementary Figure 1** The authors' production over time.

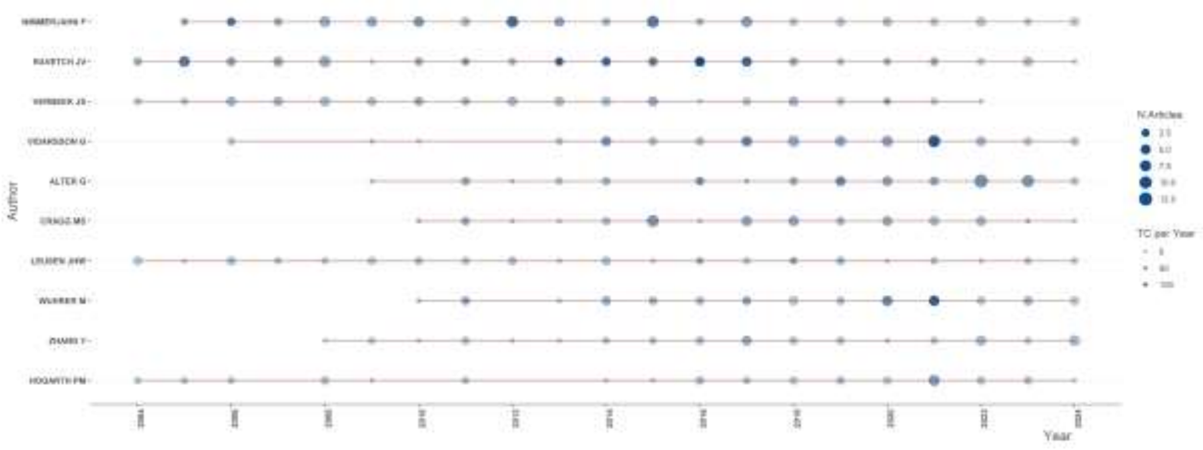


**Supplementary Figure 2** Country scientific production map

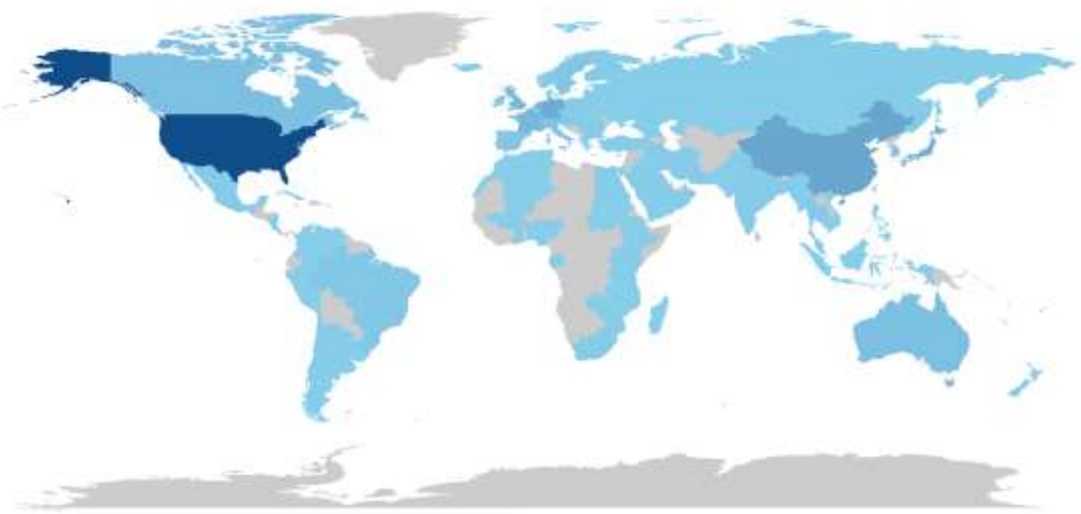

**Supplementary Figure 3** Top 25 references with the strongest citation bursts.

| References | Year | Strength | Begin | End | 2004 - 2024 |
|---|---|---|---|---|---|
| Ravetch JV, 2001, ANNU REV IMMUNOL, V19, P275, DOI 10.1146/annurev.immunol.19.1.275, DOI | 2001 | 70.22 | **2004** | 2006 | |
| Cartron G, 2002, BLOOD, V99, P754, DOI 10.1182/blood.V99.3.754, DOI | 2002 | 34.8 | **2004** | 2007 | |
| Takai T, 2002, NAT REV IMMUNOL, V2, P580, DOI 10.1038/nri856, DOI | 2002 | 32.86 | **2004** | 2007 | |
| Weng WK, 2003, J CLIN ONCOL, V21, P3940, DOI 10.1200/JCO.2003.05.013, DOI | 2003 | 28.82 | **2005** | 2008 | |
| Nimmerjahn F, 2005, IMMUNITY, V23, P41, DOI 10.1016/j.immuni.2005.05.010, DOI | 2005 | 41.71 | **2006** | 2010 | |
| Nimmerjahn F, 2006, IMMUNITY, V24, P19, DOI 10.1016/j.immuni.2005.11.010, DOI | 2006 | 63.33 | **2007** | 2011 | |
| Nimmerjahn F, 2005, SCIENCE, V310, P1510, DOI 10.1126/science.1118948, DOI | 2005 | 25.42 | **2007** | 2010 | |
| Boruchov AM, 2005, J CLIN INVEST, V115, P2914, DOI 10.1172/JCI24772, DOI | 2005 | 17.77 | **2007** | 2010 | |
| Lazar GA, 2006, P NATL ACAD SCI USA, V103, P4005, DOI 10.1073/pnas.0508123103, DOI | 2006 | 22.09 | **2008** | 2011 | |
| Nimmerjahn F, 2008, NAT REV IMMUNOL, V8, P34, DOI 10.1038/nri2206, DOI | 2008 | 108.32 | **2009** | 2013 | |
| Musolino A, 2008, J CLIN ONCOL, V26, P1789, DOI 10.1200/JCO.2007.14.8957, DOI | 2008 | 31.45 | **2009** | 2013 | |
| Zhang W, 2007, J CLIN ONCOL, V25, P3712, DOI 10.1200/JCO.2006.08.8021, DOI | 2007 | 19.51 | **2009** | 2012 | |
| Bruhns P, 2009, BLOOD, V113, P3716, DOI 10.1182/blood-2008-09-179754, DOI | 2009 | 45.95 | **2010** | 2014 | |
| Smith KGC, 2010, NAT REV IMMUNOL, V10, P328, DOI 10.1038/nri2762, DOI | 2010 | 24.78 | **2012** | 2015 | |
| Bruhns P, 2012, BLOOD, V119, P5640, DOI 10.1182/blood-2012-01-380121, DOI | 2012 | 38.73 | **2013** | 2017 | |
| Ferrara C, 2011, P NATL ACAD SCI USA, V108, P12669, DOI 10.1073/pnas.1108455108, DOI | 2011 | 27.38 | **2013** | 2016 | |
| Anthony RM, 2011, NATURE, V475, P110, DOI 10.1038/nature10134, DOI | 2011 | 22.86 | **2013** | 2015 | |
| Schwab I, 2013, NAT REV IMMUNOL, V13, P176, DOI 10.1038/nri3401, DOI | 2013 | 23.43 | **2014** | 2017 | |
| Hogarth PM, 2012, NAT REV DRUG DISCOV, V11, P311, DOI 10.1038/nrd2909, DOI | 2012 | 20.78 | **2014** | 2017 | |
| Guilliams M, 2014, NAT REV IMMUNOL, V14, P94, DOI 10.1038/nri3582, DOI | 2014 | 52.61 | **2015** | 2019 | |
| Pincetic A, 2014, NAT IMMUNOL, V15, P707, DOI 10.1038/ni.2939, DOI | 2014 | 29.99 | **2015** | 2019 | |
| DiLillo DJ, 2014, NAT MED, V20, P143, DOI 10.1038/nm.3443, DOI | 2014 | 18.06 | **2016** | 2019 | |
| Bruhns P, 2015, IMMUNOL REV, V268, P25, DOI 10.1111/imr.12350, DOI | 2015 | 30.52 | **2017** | 2020 | |
| Dekkers G, 2017, FRONT IMMUNOL, V8, P0, DOI 10.3389/fimmu.2017.00877, DOI | 2017 | 29.57 | **2018** | 2022 | |
| Bournazos S, 2020, NAT REV IMMUNOL, V20, P633, DOI 10.1038/s41577-020-00410-0, DOI | 2020 | 33.83 | **2021** | 2024 | |

**Supplementary Figure 4** The evolving trend of keywords

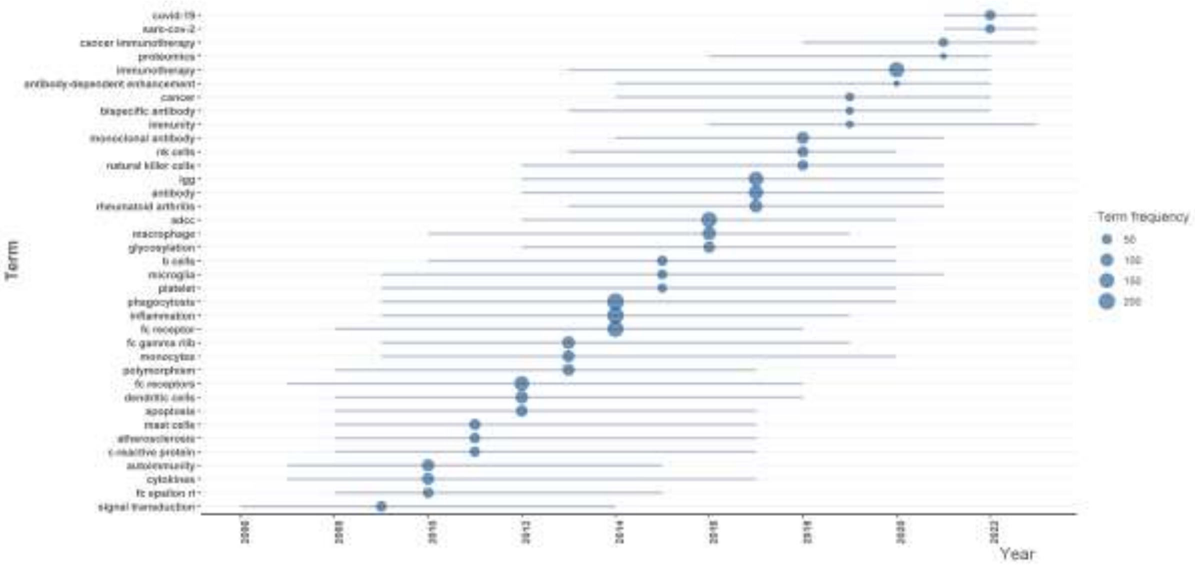

Supplement: Supplementary file 2 [file medi-104-e42695-s002.pdf]
